# Thermal Stability of Polycaprolactone Grafted Densely with Maleic Anhydride Analysed Using the Coats–Redfern Equation

**DOI:** 10.3390/polym14194100

**Published:** 2022-09-30

**Authors:** Kotchaporn Thangunpai, Donghao Hu, Xianlong Su, Mikio Kajiyama, Marcos A. Neves, Toshiharu Enomae

**Affiliations:** 1 Graduate School of Science and Technology, University of Tsukuba, 1-1-1 Tennodai, Tsukuba 305-8572, Ibaraki, Japan; 2 Faculty of Life and Environmental Science, University of Tsukuba, 1-1-1 Tennodai, Tsukuba 305-8572, Ibaraki, Japan; 3 Department of Chemistry & State Key Laboratory of Molecular Engineering of Polymers, Fudan University, Shanghai 200433, China

**Keywords:** Coats–Redfern equation, grafting, maleic anhydride, poly(ε-caprolactone), thermal stability

## Abstract

The plastic waste problem has recently attracted unprecedented attention globally. To reduce the adverse eff ects on environments, biodegradable polymers have been studied to solve the problems. Poly(ε-caprolactone) (PCL) is one of the common biodegradable plastics used on its own or blended with natural polymers because of its excellent properties after blending. However, PCL and natural polymers are difficult to blend due to the polymers’ properties. Grafted polymerization of maleic anhydride and dibenzoyl peroxide (DBPO) with PCL is one of the improvements used for blending immiscible polymers. In this study, we first focused on the effects of three factors (stirring time, maleic anhydride (MA) amount and benzoyl peroxide amount) on the grafting ratio with a maximum value of 4.16% when applying 3.000 g MA and 1.120 g DBPO to 3.375 g PCL with a stirring time of 18 h. After that, the grafting condition was studied based on the kinetic thermal decomposition and activation energy by the Coats–Redfern method. The optimal fitting model was confirmed by the determination coefficient of nearly 1 to explain the contracting volume mechanism of synthesized PCL-g-MA. Consequently, grafted MA hydrophilically augmented PCL as the reduced contact angle of water suggests, facilitating the creation of a plastic–biomaterial composite.

## 1. Introduction

Benefiting from their outstanding physical and chemical properties, such as flexibility, light weight and low cost, plastics have played an extremely important role in modern society [1,2,3,4,5]. However, with more and more common nondegradable types of plastics (such as polyethylene (PE), polypropylene (PP), polystyrene (PS) and polyvinyl chloride (PVC)) being produced, unrecycled plastic waste has become a crucial problem all over the world [6,7,8,9,10], causing many environmental and health problems [11,12,13,14,15,16]. According to the reports from the Organization for Economic Co-operation and Development (OECD), 6300 mega tons of plastics was produced between 1950 and 2015, while more than 80% of those were accumulated in landfills or left in the natural environment [17]. These problems were affected by the recalcitrant nature that makes the plastic waste unyielding to biodegradation due to its longer duration of degradation time [18]. Since the early 1970s, many studies and regulations related to plastic waste issues have been carried out to solve this problem [19,20,21]. One of these to focus on and to try to develop is biodegradable polymers. Biodegradable polymers can be classified into two main groups: natural polymers (e.g., polysaccharides, proteins and nucleic acids) and synthetic polymers (e.g., polyester: polycaprolactone and poly (amino acids)) [22,23,24].

Poly(ε-caprolactone) (PCL) is a biodegradable semi-crystalline synthetic polyester with excellent biocompatibility, flexibility and thermo-plasticity properties [25]. Various applications of it and relevant copolymers have been reported, such as drug delivery systems [26,27,28,29,30] and adhesives for bookbinding and packaging [31,32,33]. PCL can be degraded within several months to several years depending on the molecular weight, natural environment conditions, pH, purity, thickness and so on [34,35,36]. Attempts have also been made to blend PCL widely with natural polymers such as polysaccharides that constitute paper materials and proteins to improve the properties of biodegradable polymers. However, the two different kinds of polymers are rather immiscible due to their lack of affinity [37]. To improve the blending affinity of immiscible polymers, graft polymerization is commonly applied as an effective method of modifying the physicochemical interactions and phase dispersion between the blending components. Graft polymerization can be induced by the attaching of monomers to the surface of polymers through covalent bonds [38] with suitable unsaturated polar functional groups, such as amines, and anhydrides [39,40].

A maleated component is a functional site usually consisting of some polar groups to increase interfacial adhesion and/or interaction between two or more components [41]. Representatives are maleic anhydride (MA), glycidyl methacrylate, acrylic acid and oxazoline. The most preferred is MA from the aspect of the facilitated modification of polymers since it is easy-to-handle, low toxic, not likely to be homopolymerized during a mild free radical melt-grafting and highly miscible when the components are combined [41,42]. The reaction of MA groups can be produced in the presence of a peroxide radical initiator through a grafting reaction in any of the melt phases, solid state, suspension/emulsion and solution. Among them, the solution grafting proceeds in a solvent for suitably dissolving a reactant polymer and provides high grafting ratios without degradation of the polymer during the reaction [43,44]. In addition, a peroxide radical initiator is required to exert a crucial effect on the grafting polymer process. In the grafting processes, the initial reaction induced by the initiator is the dehydrogenation of hydrogen atoms from α-carbon atoms to the ester carbonyl groups to form polymeric macroradicals. Then, the end of the radical chain also forms the macromer molecules via β-scissoring [45]. Dibenzoyl peroxide (DBPO) is one of the initiators widely used for grafting reactions because of its slow initiator decomposition rate at a suitable reaction temperature [41].

The thermal stability of a biodegradable polymer is one of the essential factors reflecting their degradation feasibility [46]. The thermal response and combustion characteristics obtained from important reaction kinetic parameters, such as activation energy, reaction order and pre-exponential factor, can be applied to exhibit the thermal stability and give guidance for the synthesis of biodegradable polymers [47]. To determine, calculate and explain the decomposition mechanisms from the aspect of the study of solid-state kinetics, the reaction models of the monomer, polymer or copolymer before and after blending have been studied. Kinetic studies have been conducted using thermal analysis (TGA/DTA) to estimate the thermal stability of the polymer. The Coats–Redfern (CR) kinetic model is a common integral method and has been applied to the studies of the thermal decomposition of coal and biomass [48,49,50]. The TGA/DTA data (weight loss, heating rate, etc.) was used to evaluate the kinetic parameters of solid-state reactions. The accurate and precision temperature measurement was conducted and calculated from a linear heating rate because of endo- or exo-thermic reactions [51].

In this research, we focused on an optimum condition for the synthesis of PCL grafted with MA (PCL-g-MA) with the highest possible grafting ratio. Consequently, the highest grafting ratio helps explain the thermal decomposition model and activation energy using the CR model. In addition, the highest grafting ratio is expected to provide the highest hydrophilicity to the plastic so that the surface is hydrophilically augmented to be more miscible with hydrophilic materials such as paper and other biomass.

## 2. Materials and Methods

### 2.1. Materials

PCL (M_w_ = 10,000 g/mol), DBPO, MA (M_w_ = 98.06 g/mol), tetrahydrofuran (THF), hexane, potassium hydroxide (KOH), ethanol (C_2_H_5_OH) and phenolphthalein ethanol solution (1.0% *w*/*v*) were purchased from Fujifilm Wako Pure Chemical (Osaka, Japan). A filter paper (No.4, Monotaro, Kiriyama, Japan) was utilized for the experiment.

### 2.2. Synthesis of PCL-g-MA

**Scheme 1:**
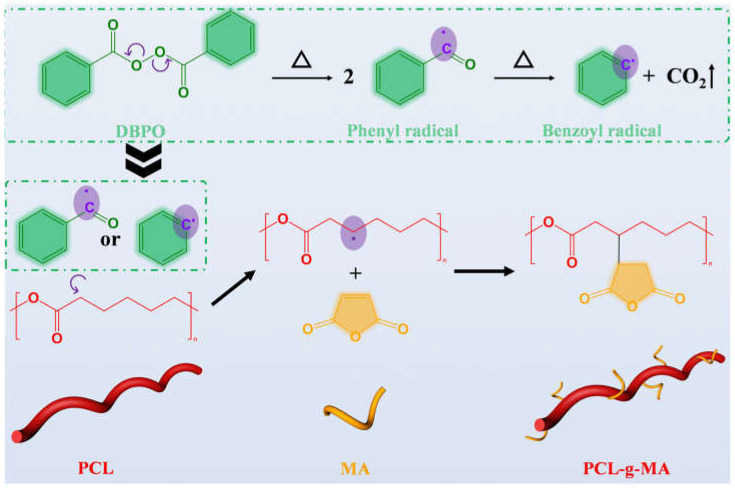
Grafting reaction of MA onto PCL.

The synthesis of MA-grafted PCL (PCL-g-MA) was modified based on a previous study [52]. Benzoyloxy radicals were produced by the homolysis of DBPO. Then, DBPO-free radicals attack the PCL molecule to generate PCL radicals. Finally, MA moieties react to the PCL radicals (Scheme 1).

In the experiment, 3.375 g PCL and a mass of MA specified in following tables were weighed precisely and dissolved in 40 mL THF at 40 ± 2 °C for 30 min in an oil bath under an N_2_ atmosphere. DBPO was then added and stirred for various periods. The condition was grouped into three factors in Table 1, Table 2 and Table 3.

After that, the THF solution of PCL, DBPO and MA was poured into hexane (1:10 *v*/*v*) and was gently stirred to precipitate the compounds at 25 °C for 10 min. Finally, the precipitated polymer was filtrated and dried in a vacuum desiccator at 25 °C. For each of the series, an optimum condition was screened.

### 2.3. Characterization

#### 2.3.1. Fourier Transform Infrared Spectroscopy (FTIR)

The functional groups of the samples were identified with an FTIR spectrophotometer (FT/IR-6100, JEOL, Tokyo, Japan) in the range of 500–4000 cm^−1^. Subsequently, pellets of the PCL-g-MA specimens mixed with KBr (1:100 *w*/*w*) were prepared, dried in a desiccator for 24 h and subjected to the FTIR measurement.

#### 2.3.2. X-Ray Photoelectron Spectroscopy (XPS)

The surface compositions of the specimens were further analysed by X-ray photoelectron spectroscopy (XPS) (JPS-9010TR, JEOL, Tokyo, Japan) with an AlKα radiation (1486.6 eV) source at 10 kV and 20 mA.

#### 2.3.3. Determination of Grafting Ratio

A total of 2 g of the synthesized polymer was heated in 200 mL THF at 40 ± 2 °C for 2 h. Subsequently, the heated solution was immediately titrated with a 0.03 M ethanolic KOH solution with phenolphthalein as an indicator. The MA loading percentage in the solution was applied to calculate the acid number and grafting ratio by Equations (1) and (2) [53]:

(1) $$Acid\;number\;\left(\mathrm{mgKOH}/g\right)=\frac{C_{KOH}\left(M\right)\times V_{KOH}\left(\mathrm{mL}\right)\times 56.1}{m\left(\mathrm{polymer}\left(g\right)\right)},$$

where *C_KOH_* is the concentration of ethanolic KOH solution (mol∙L^−1^), *V_KOH_* is the PCL-g-MA titrate volume (mL), *m* (polymer) is the mass of copolymer (g).

(2) $$Grafting\;ratio\;\left(\%\right)=\frac{Acid\;number\times 98.1}{2\times 561}.$$

#### 2.3.4. Field Emission Scanning Electron Microscope (FESEM)

Samples were coated with platinum (Pt) using a spatter before the observation with a field emission scanning electron microscope (SU8020, Hitachi, Tokyo, Japan).

#### 2.3.5. Contact Angle Measurement

Contact angle (CA) measurement was carried out with PCL and PCL-g-MA coated on a glass surface by a sessile drop method (DropMaster DMs-401, Kyowa Interface Science, Saitama, Japan). Deionized water with a volume of approx. 20 μL was dropped on the surface of them. The measurement was conducted at the interface between air and deionized water.

#### 2.3.6. Thermal Property Measurement (TGA/DTG/DSC)

Thermogravimetric analysis (TGA) was conducted with a thermogravimetric analyser (TG/DTA7300, Seiko Instruments Inc., Chiba, Japan) and heated up to 550 °C at a heating rate of 15 °C/min and a flow rate of 200 mL/min of argon gas. The differential scanning calorimetry (DSC) (X-DSC-7000, Shimadzu, Kyoto, Japan) curves were determined during heating (10–110 °C) and cooling (110 to −20 °C) processes under the same atmosphere. The crystallinities (Xc) of PCL and each synthesized PCL-g-MA were calculated using the standard enthalpy of PCL (ΔHm = 139.5 J/g) [54]. Xc was calculated based on the ratio of the enthalpy of synthesized PCL-g-MA to that of pure PCL.

#### 2.3.7. The Coats–Redfern Equation [51]

The effect of an absorbed dose on the thermal stability of PCL-g-MA was evaluated from the aspect of the thermal properties obtained by TGA and DTA. The absorbed doses were varied by changing the heating rate of TGA and DTA from 5–30 °C/min in each condition.

Theoretical analysis

The rate of a solid-state reaction can be represented by the Arrhenius plot expressed from Equation (3):

(3) $$\frac{d\alpha }{dt}=A\;e^{-\frac{E_{a}}{RT}}f\left(\alpha \right),$$

where *A* is the pre-exponential factor, *E_a_* is the activation energy, *T* is the absolute temperature, R is the gas constant, *f*(*α*) is the differential conversion function and *α* is the conversion fraction from the gravimetric measurement, which is defined as Equation (4) [55]:

(4) $$\alpha =\frac{m_{0}-m_{t}}{m_{0}-m_{f}},$$

where *m*_0_, *m_t_* and *m_f_* are the initial weight, the weight at a given time *t* and the final weight, respectively.

In general, the kinetic analysis is conducted using the iso-conversional method, which assumes that the reaction rate is a function of only temperature as the independent variable and can be classified into two sub-methods.

Isothermal method

Equation (5) is obtained by integrating the rate under isothermal conditions:

(5) $$\ln\left(t\right)=\ln\left[\frac{\;g\left(\alpha \right)}{A}\right]+\frac{E_{a}}{RT},$$

where *g*(*α*) is the integral conversion function defined as Equation (6):

(6) $$g\left(\alpha \right)=\int_{0}^{\alpha }\frac{d\alpha }{f\left(\alpha \right)},$$

From Equation (5), *E_a_* and the constant ln[*g(α)*/*A*] can be obtained from the slope and the intercept of the linear relationship between ln(*t*) and *T*^−1^ for *α* = constant.

Nonisothermal method

The reaction rate can be represented in the nonisothermal condition as Equation (7):

(7) $$g\left(\alpha \right)=\frac{A}{\beta }\int_{0}^{T}e^{-\left(\frac{E_{a}}{RT}\right)}dT,$$

where *β* is the heating rate.

By using the Coats–Redfern equation to solve Equation (7) when 2RT/E ≪ 1, the reordered equation can be rewritten as Equation (8):

(8) $$\ln\frac{g\left(\alpha \right)}{T^{2}}=\ln\left(\frac{AR}{\beta E}\right)-\frac{E_{a}}{RT}.$$

The TGA data of the maximum grafting ratio of PCL-g-MA were calculated by using the Coats–Redfern Equation to study the synthesis process model. The linear regression is represented by ln[*g(α)*/*T*^2^] as a function of *T*^−1^ to determine *E_a_* and *A* from the slope and the intercept, respectively. The algebraic expressions of *f*(*α*) and *g*(*α*) for the kinetic models are listed in Table 4 [56,57,58,59].

## 3. Results and Discussion

### 3.1. Infrared Spectroscopy (FTIR)

**Figure 1:**
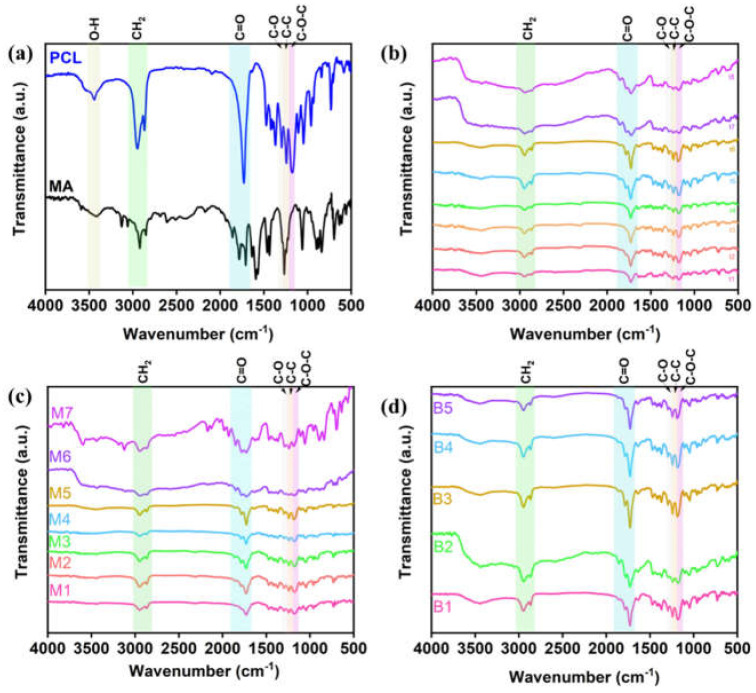
FTIR spectra of (**a**) PCL and MA, and PCL-g-MA synthesized with different (**b**) reaction periods, (**c**) amounts of MA and (**d**) amounts of DBPO.

Figure 1 shows the FTIR spectra of PCL, MA and PCL-g-MA synthesized with the mentioned different experimental conditions. PCL showed the characteristic peak of hydroxyl groups at 3444 cm^−1^. Two distinct peaks at 2952 and 2869 cm^−1^ corresponded to the asymmetrical and symmetrical stretching of CH_2_, respectively. As for MA, the C=O peaks of MA were found at 1861 (low intensity) and 1785 cm^−1^ (medium intensity) due to the C=O stretching group of low and high intensity hydrolysed MA, respectively [60]. PCL-g-MA was successfully generated by grafting MA onto PCL since two characteristic shoulders of anhydride carboxyl groups appeared at 1790 and 1867 cm^−1^ [61]. Additionally, the C-O and C-C stretchings were located at 1298 and 1244 cm^−1^, respectively, while the combination asymmetric C-O-C stretching and the symmetric C-O-C stretching peak was located at 1179 cm^−1^ [61,62]. In all spectra, the C=O group peaks were present at 1730 cm^−1^. The above results confirmed a similar tendency to the previous reports [63,64] that MA was successfully grafted onto PCL.

### 3.2. XPS

**Figure 2:**
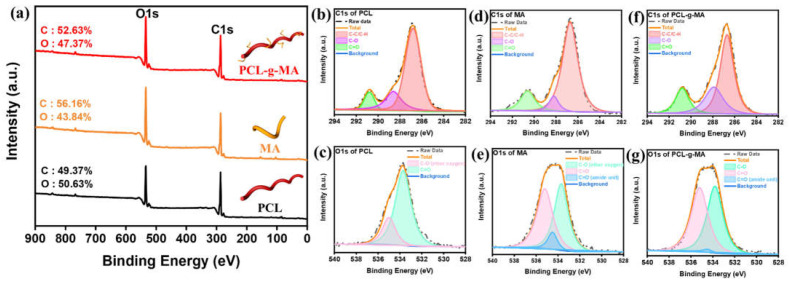
(**a**) XPS full spectra of PCL, MA and PCL-g-MA, and high-resolution XPS spectra of (**b**) C_1s_ of PCL, (**c**) O_1s_ of PCL, (**d**) C_1s_ of MA, (**e**) O_1s_ of MA, (**f**) C_1s_ of PCL-g-MA and (**g**) O_1s_ of PCL-g-MA prepared by 3.375 g PCL, 3.000 g MA and 1.120 g DBPO by 18 h.

The surface physicochemical properties were further confirmed by XPS analysis. Figure 2a shows the XPS full spectra of PCL, MA and PCL-g-MA with two corresponding peaks, C_1S_ (approx. 287 eV) and O_1s_ (approx. 534 eV), along with their relative atomic composition ratios. Figure 2b–g shows the C_1s_ and O_1s_ spectra of PCL, MA and PCL-g-MA. The C_1s_ spectrum of PCL in Figure 2b indicates three groups, including -CHx (284.5 eV), -C-O- (286.3 eV) and -C(=O)-O- (288.5 eV) [65]. The O_1s_ peaks (Figure 2c) are identified at 533.7 and 535.0 eV, corresponding to -C=O and -C-O-, respectively. The C_1s_ spectrum of MA (Figure 2d) is in accordance with the same three kinds of groups, -CHx (286.7 eV), -C-O- (288.2 eV) and -C(=O)-O- (290.6 eV). The three peaks consisting of the O_1s_ peak of MA (Figure 2e) appear at 533.8, 535.2 and 534.5 eV, representing the ether oxygen and carbonyl oxygen both in maleic anhydride and the carbonyl bond in the amide unit [66,67], respectively. The C_1s_ and O_1s_ peaks of PCL-g-MA are located at and corresponded to the same kinds of groups as those of PCL and MA (Figure 2f,g). Moreover, the O_1s_ peaks of PCL-g-MA are carbonyl groups of amide units of MA. This also proves that MA was successfully grafted on the surface of PCL. More detailed information is supplied in the supplementary material(Figures S6–S8).

### 3.3. Determination of Grafting Ratio

**Figure 3:**
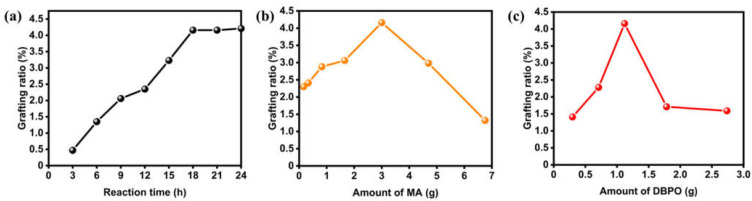
Grafting ratios of PCL-g-MA synthesized with different (**a**) reaction periods, (**b**) amounts of MA and (**c**) amounts of DBPO.

Figure 3a shows the grafting ratios of PCL-g-MA with different reaction periods. From the results, the grafting ratio obviously increased from 3 to 18 h, and attained an equilibrium state after 18 h. Figure 3b shows that the grafting ratio increased with the increase in MA amount from 0.165 to 3.000 g, while the reaction period (18 h) and initiator DBPO amount (1.120 g) were kept constant. With the increase in the added MA amount, the chance of PCL reacting with MA became higher [41,68]. In addition, with excess MA (>3.000 g) adopted for the synthesis, the grafting ratio decreased with the increasing MA. This is because overloaded MA could cause the free radicals to undergo a termination or combination reaction with PCL macroradicals, along with some unexcepted reactions (such as cage effect), all of which could lead to the reduction in the grafting ratio [69]. Figure 3c shows that the grafting ratio of PCL-g-MA first increased and then decreased with the increasing amount of DBPO (0.0297–1.120 g), which mainly resulted from the increased formation of the higher amount of DBPO that would also help raise the probability of MA transfer to the PCL backbone, resulting in a higher grafting ratio result. However, with the continuous addition of DBPO (>1.120 g), the grafting ratio drastically decreased. On the one hand, an excess of radical initiators has free radical species that can promote a termination or a combination reaction between polymer macroradicals. On the other hand, the excess radical initiators may cause the recombination of polymer radicals due to the formation of the crosslinking structure by carbon–carbon crosslinks and the depolymerization reflected by the acute reduction in molecular weight [70]. Above all, the optimum amount of DBPO, amount of MA and the reaction time for PCL-g-MA synthesis were determined to be 1.120 g, 3.000 g and 18 h, respectively.

### 3.4. Morphological Observation

**Figure 4:**
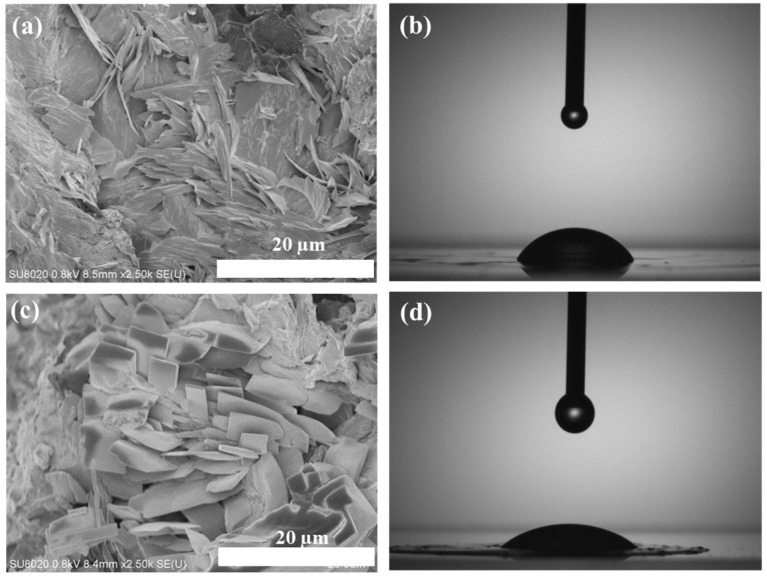
FESEM images of (**a**) PCL and (**b**) CA of water on PCL as well as FESEM images in (**c**) and (**d**) that on PCL-g-MA synthesized from 3.375 g PCL, 3.000 g MA and 1.120 g DBPO for 18 h.

Figure 4a,c show FESEM images featuring the morphology before and after grafting, where a crystallinity (X_c_) decrease could be clearly observed. It can be seen from Figure 4a that PCL has mainly fibrous and needle-like crystal structures compared to Figure 4c, PCL-g-MA, that seems to be irregular in shape and has a denser appearance on the surface. The denseness of the surface might result from the bonding between MA and PCL [71]. Meanwhile, the CA results of PCL (Figure 4b) and the synthesised PCL-g-MA (Figure 4d) were 49.9° and 40.4°, respectively. Compared to pure PCL, the surface hydrophilicity and wettability of the PCL-g-MA was enhanced prominently. This property would be expected to facilitate the mixture of PCL-g-MA and cellulosic fibers constituting paper and the creation of biodegradable plastic and paper composites.

### 3.5. DSC

**Figure 5:**
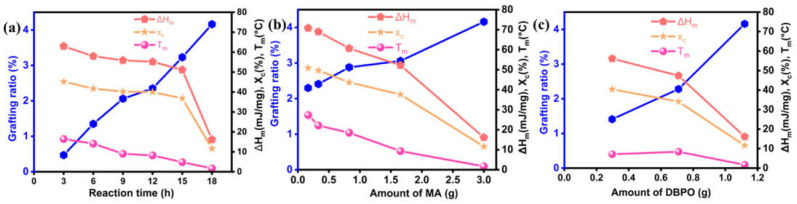
Influence of (**a**) reaction time, (**b**) amounts of MA and (**c**) amounts of DBPO on grafting ratio, T_m_, ΔH_m_ and X_c_ of synthesized PCL-g-MA.

The three parameters derived from the DSC data, the melting temperature (T_m_), the enthalpy of melting (ΔH_m_) and the X_c_ were studied to understand the influence of the reaction period, MA amount and DBPO amount on the thermal property of PCL-g-MA. Figure 5 summarizes these parameters when utilizing the reaction period, MA amount and DBPO amount of no more than their individual optimal values. T_m_, ΔH_m_ and X_c_ decreased with increasing grafting ratio and increasing time. Among them, X_c_ tended to increase, proving the results obtained from the FESEM images. The grafting ratio refers to the monomers that could be bonded with the covalent bond onto the polymer [72]. Thus, X_c_ of PCL-g-MA is presumed to be influenced by the addition of MA, which should reduce the degree of crystallinity because MA interacted with plain PCL and inhibited the crystal growth [73]. Figure 5b suggests that T_m_, ΔH_m_ and X_c_ decreased with an increasing MA amount of addition so that more MA were grafted on principal PCL chains [2]. Figure 5c shows that T_m_, ΔH_m_ and X_c_ decreased with the increasing amount of DBPO. The increased amount of DBPO increased the MA grafting probability, and then decreased ΔH_m_, T_m_ and X_c_ of the synthesised PCL-g-MA as compared with those of pure PCL [74]. The reaction time, MA amount and DBPO amount had an influence on the grafting ratio, T_m_, ΔH_m_ and X_c_. It is presumed that the higher the latter three parameter values, the lower the grafting ratio.

### 3.6. Theoretical Analysis Using the Coats–Redfern Method

**Figure 6:**
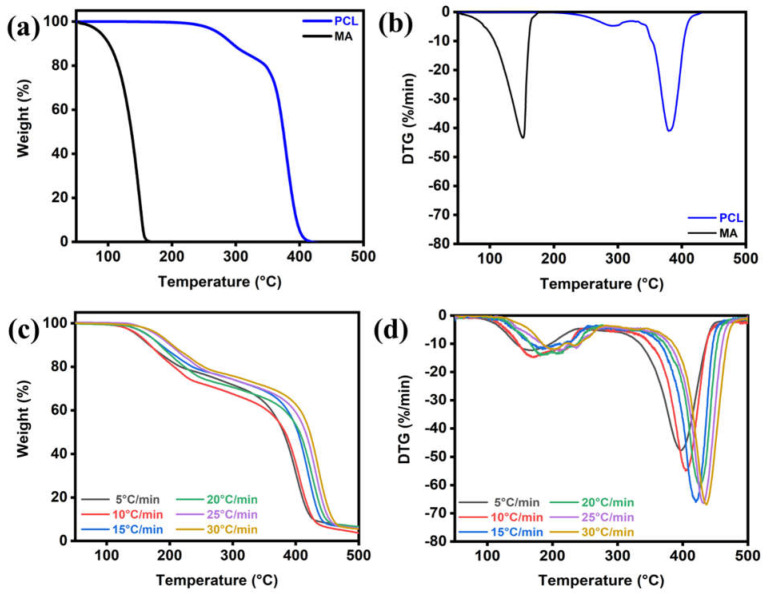
(**a**) Thermograms and (**b**) DTG curves of PCL and MA, and (**c**) thermograms and (**d**) DTG curves of PCL−g−MA at a maximum grafting ratio all at various heating rates.

Figure 6a,b show the TG and DTG curves of pure PCL and MA. Plain PCL shows a large peak in the temperature range of 280–430 °C with a maximum degradation temperature (*T_max_*) at 378 °C due to heat degradation [75]. Pure MA exhibited a mass reduction in the temperature range 62–175 °C with a *T_max_* at 149 °C relisted to its vaporization [76]. The pure PCL shows two steps of TGA and DTG in Figure 6a,b; the first mass loss (approximately 10%) curve with an onset degradation temperature around 290 °C was observed due to the range of PCL at a low molecular weight (short chains) [77]. The major and sharp weight loss of pure PCL occurring in the temperature range of 320–437 °C is attributed to a large-scale thermal degradation of the PCL chains [78]. As for PCL-g-MA (Figure 6c,d) at a heating rate of 15 °C/min it exhibits two weight loss steps corresponding to MA degradation (106–188 °C) and PCL degradation (282–430 °C).

**Figure 7:**
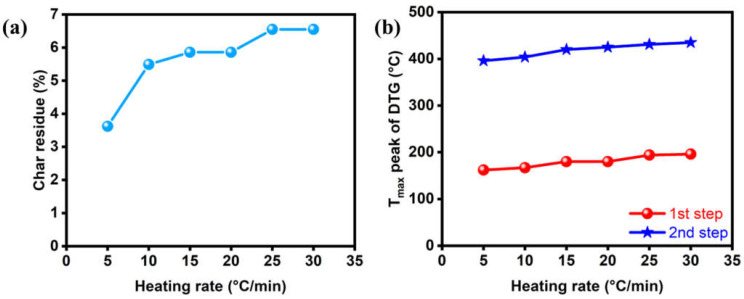
(**a**) Char residue at 550 °C and (**b**) *T_max_* peak of DTG of PCL-g-MA at a maximum grafting ratio and degradation temperature as a function of heating rate.

In addition, to understand the applicability of the C-R model, TG curves and char residual at 550 °C of a decomposition process for the optimum synthesised PCL-g-MA at different heating rates of 5, 10, 15, 20, 25 and 30 °C/min are compared in Figure 6c, and the DTG curves for the optimum condition to synthesise PCL-g-MA at heating rates of 5, 10, 15, 20, 25 and 30 °C/min are compared in Figure 6d. From the results of TGA and DTG in Figure 6c,d, the thermal decomposition of PCL-g-MA can be divided into two main stages. The first stage indicates MA vaporization at a lower temperature than PCL. Then, the second stage corresponds to the thermal degradation of PCL. All the results of the thermogram characterization of PCL-g-MA initial temperature (*T_i_*), *T_max_*, final temperature (*T_f_*) and residue at 550 °C of a decomposition process obtained at different heating rates are summarized in Table S9 (supplementary material part). The above results indicate that with an increasing heating rate, both *T_i_* and *T_max_* increased [79]. While there was a small amount of residual ash for PCL-g-MA above 500 °C, there was no residual found in pure PCL and MA. This means that the grafted MA improves the thermal stability of PCL [80]. Thus, *T_max_* exists in the range from 396 to 435 °C and seems to reflect the PCL structure. All PCL-g-MA samples underwent less weight loss than PCL because the MA portion tends not to degrade. The further information corresponding to the TGA curves of PCL and PCL-g-MA (Figure 6a,c) is summarized in Table S9 (supplementary material part). It is observed that *T_max_* of pure PCL was only 378 °C, while PCL-g-MA showed a higher *T_max_* of 420 °C. The higher *T_max_* obtained with PCL-g-MA indicates that the MA grafted on the PCL chain improves the thermal stability of the copolymer [80]. The tendencies of the char residue at 550 °C and at maximum degradation temperature at various heating rates are shown in Figure 7a,b. The percentage of char residue increased from 3.62% to 6.55% with an increase in the heating rate.

The relationships between ln(*g*(*α*)/*T*^2^) and *T*^−1^ according to Equation (8) using differential function g(α) from Table 4 are shown in Table 5. The kinetic models were chosen from the linear approximation to be with a high determination coefficient (R^2^). Table 5 shows model R3 provided the linear trend with a higher determination coefficient (R^2^ equals approx. 0.99) than the other models with α from TGA-simulated curves with the spherical symmetry based on the Geometrical Contraction Model (R3) based on the Coats–Redfern method for the reaction of the modification of PCL-g-MA. These models and results indicate the pyrolysis of the PCL-g-MA structure so that the positions of MA grafted on a PCL main chain are more likely to undergo random scissions first due to the weak bonding from C-C structure to produce shorter monomers and generate free radicals of the polymer. After that, the PCL structure starts to degrade by random scissions via cis-elimination reactions and progresses with cyclic rupture via intramolecular transesterification at over 300 °C [74,81]. The pyrolysis results confirmed the same tendency as suggested by the TGA and DTA results in Figure 6.

**Figure 8:**
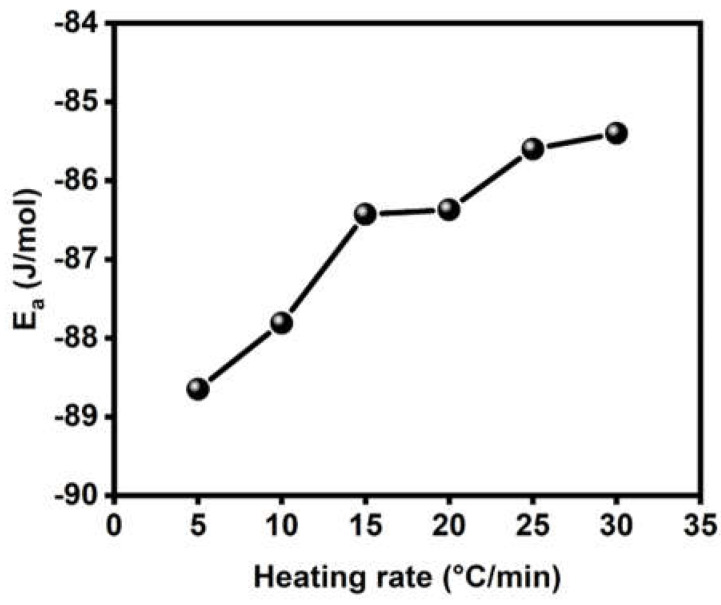
Activation energies calculated based on the contracting volume model (R3) at various heating rates.

Consequently, model R3 was the most suitable model to explain the thermal decomposition of the synthesized PCL-g-MA, where the *E_a_* of model R3 was calculated and is shown in Figure 8. The *E_a_* is expressed in the exothermal reaction of the modification of PCL-g-MA (detailed information in supplementary material Table S10). *E_a_* is the parameter that is represented to be evaluated from the thermogravimetric analysis because the thermal breakage of the chemical bonds of the polymer provided the solid–gas conversion of the biomass in combustible gas, and *E_a_* may also provide the needed energy information for starting the chemical decomposition. Thus, the exothermal reaction could be related to the breakage of the C–C bonds of the polymers [82].

## 4. Conclusions

An optimum condition to synthesise PCL-g-MA with the highest grafting ratio was determined by the measurement of FTIR spectra, which provided peaks at 1790 and 1867 cm^−1^ attributed to maleic anhydride. This result indicates a successful grafting of MA on PCL surfaces. Factors involved with the high grafting ratio were the amount of DBPO and MA, and the reaction period. The optimum condition was 1.120 g and the amount of MA added 3.000 g for a reaction time of 18 h. Additionally, the results of the contact angle also confirmed the relationship between the grafting ratio and the hydrophilic property. The hydrophilic property was increased by the grafting of MA on PCL. The thermal stability of PCL-g-MA decreased with the increasing amount of grafted MA. The PCL-g-MA synthesised at an optimum condition was analysed using TGA at various heating rates from 5–30 °C/min at an interval of 5 °C/min, and then all the TGA data were explained with the Coats–Redfern equation to find the best fitting kinetic model. The results of the TGA analysis were plotted as ln(*g*(*α*)/*T*^2^) as a function of 1/*T* for different kinetic models using different values of g(α). The highest determination coefficient R^2^ was obtained when applying a linear regression based on the spherical symmetry diffusion mechanism (R3), suggesting this model is suitable for explaining the synthesis of PCL-g-MA. After that, *Ea* was calculated and expressed in the exothermal reaction of the modification of PCL-g-MA that explained the relation of the breaking of the carbon–carbon bonds of the polymers.

## Figures and Tables

**Table 1:** Experimental design for the factor of stirring time.

Sample	Time (h)	Amount of DBPO (g)	Amount of MA (g)
t1	3	1.120	3.000
t2	6	1.120	3.000
t3	9	1.120	3.000
t4	12	1.120	3.000
t5	15	1.120	3.000
t6	18	1.120	3.000
t7	21	1.120	3.000
t8	24	1.120	3.000

**Table 2:** Experimental design for the factor of the addition amount of MA.

Sample	Time (h)	Amount of DBPO (g)	Amount of MA (g)
M1	18	1.120	0.165
M2	18	1.120	0.331
M3	18	1.120	0.827
M4	18	1.120	1.655
M5 (t6)	18	1.120	3.000
M6	18	1.120	4.500
M7	18	1.120	6.754

**Table 3:** Experimental design for the factor of the addition amount of DBPO.

Sample	Time (h)	Amount of DBPO (g)	Amount of MA (g)
B1	18	0.297	3.000
B2	18	0.709	3.000
B3 (t6)	18	1.120	3.000
B4	18	1.784	3.000
B5	18	2.738	3.000

**Table 4:** Thermal kinetic models for solid-state kinetics with expression for *f*(*α*) and *g*(*α*).

Mechanism Model	Differential Form *f*(*α*)	Integral Form *g*(*α*)
Order of Reaction		
First order (F1)	1 − α	−ln(1 − α)
Second order (F2)	(1 − α)^2^	(1 − α)^−1^ − 1
Third order (F3)	(1 − α)^3^	[(1 − α)^−2^ − 1]/2
Diffusion Models		
One-dimensional diffusion (D1)	1/2α	α^2^
Two-dimensional diffusion (D2)	[−ln(1 − α)]^−1^	[(1 − α)ln(1 − α)] + α
Three-dimensional diffusion, Jandel (D3)	3(1 − α)^2/3^/[2(1 − (1 − α)1/3)]	[1 − (1 − α)^1/3^]^2^
Diffusion control, Ginstling–Brounshtein (D4)	3/2[(1 − α)^−1/3^ − 1]	1 − (2α/3) − (1 − α)^2/3^
Nucleation Growth Models		
Two-dimensional (A2)	2(1 − α)[-ln(1 − α)]^1/2^	[−ln(1 − α)]^1/2^
Three-dimensional (A3)	3(1 − α)[-ln(1 − α)]^3/2^	[−ln(1 − α)]^1/3^
Four-dimensional (A4)	4(1 − α)[-ln(1 − α)^3/4^	[−ln(1 − α)]^1/4^
Geometrical Contraction Model		
One dimension (R1)	1	A
Two dimensions (R2)	2(1 − α)]^1/2^	1 − (1 − α)^1/2^
Three dimensions (R3)	2(1 − α)]^1/2^	1 − (1 − α)^1/3^
Experimental Nucleation Models		
Power law (P2)	2α^1/2^	α^1/2^
Power law (P3)	3α^2/3^	α^1/3^
Power law (P4)	4α^3/4^	α^1/4^

**Table 5:** Determination coefficient R^2^ calculated from kinetic parameters for different models with α values obtained from TGA-simulated curves by the Coats–Redfern equation.

Mechanism Models	Heating Rate (°C/min)					
	5	10	15	20	25	30
Order of Reaction						
First order (F1)	0.8780	0.7861	0.5476	0.3249	0.1594	0.0849
Second order (F2)	0.8058	0.7271	0.7021	0.6985	0.6826	0.7069
Third order (F3)	0.6923	0.5948	0.5856	0.5562	0.5722	0.5719
Diffusion Models						
One-dimensional diffusion (D1)	0.9581	0.6746	0.3735	0.1831	0.0689	0.0293
Two-dimensional diffusion (D2)	0.8877	0.9329	0.8959	0.9465	0.8998	0.9338
Three-dimensional diffusion, Jandel (D3)	0.9200	0.8251	0.7380	0.7189	0.6116	0.5684
Diffusion control, Ginstling–Brounshtein (D4)	0.8956	0.8368	0.8131	0.8015	0.7905	0.7839
Nucleation Growth Models						
Two-dimensional (A2)	0.8800	0.8953	0.8588	0.9038	0.8400	0.8935
Three-dimensional (A3)	0.8800	0.8953	0.8588	0.9038	0.8400	0.8935
Four-dimensional (A4)	0.8800	0.8953	0.8588	0.9038	0.8400	0.8935
Geometrical Contraction Model						
One dimension (R1)	0.7936	0.8000	0.5663	0.3236	0.1403	0.0639
Two dimensions (R2)	0.9683	0.9282	0.8875	0.8803	0.8277	0.8037
Three dimensions (R3)	0.9949	0.9888	0.9839	0.9822	0.9747	0.9706
Experimental Nucleation Models						
Power law (P2)	0.7936	0.8000	0.5663	0.3236	0.1403	0.06386
Power law (P3)	0.7936	0.8000	0.5663	0.3236	0.1403	0.06386
Power law (P4)	0.7936	0.8000	0.5663	0.3236	0.1403	0.06386

## Data Availability

The data presented in this study are available on request from the corresponding authors.

## Supplementary Materials

The following supporting information can be downloaded at: https://www.mdpi.com/article/10.3390/polym14194100/s1, Figure S1: Thermograms of PCL-g-MA; Figure S2: Derivative thermogravimetric analysis (DTG) of PCL-g-MA; Figure S3: Differential scanning calorimetry (DSC) thermograms; Figure S4: X-ray Diffraction (XRD) of (a) PCL and MA, PCL-g-MA; Figure S5: Nuclear Magnetic Resonance (NMR) of (a) PCL, (b) MA, and (c) PCL-g-MA [83,84]; Figure S6: High resolution XPS spectra for C1s and O1s of various reaction time; Figure S7: High resolution XPS spectra for C1s and O1s of various MA added; Figure S8: High resolution XPS spectra for C1s and O1s of various DBPO added; Table S1–S4: Summary of DSC measurement results; Table S5–S8: Summary of the high-resolution results; Table S9: Summary of the *T_i_*, *T_max_*, *T_f_*, DTG at main *T_max_* peak and residue at 550 °C at the different heating rates; Table S10: Activation energy calculated based on the contracting volume model (R3) at various heating rates.
