# Supplementary material for: Thermal Stability of Polycaprolactone Grafted Densely with Maleic Anhydride Analysed Using the Coats–Redfern Equation
Source: Polymers (Basel). 2022 Sep 30;14(19):4100. doi: 10.3390/polym14194100 (PMC9571202; doi:10.3390/polym14194100)
Supplement: Supplementary file 1 [file polymers-14-04100-s001.zip › polymers-1918108-supplementary.pdf]

polymers

MDPI

Supplementary materials

1. Materials and Methods

1.1. X-ray diffractometry (XRD)

The crystal structure was analyzed using X-ray diffractometry (XRD) (D8 Advanced, AXS-Bruker, Germany) in a 2θ range from 10° to 60° at 40 kV and 40 mA of acceleration using Cu-Kα radiation (1.5418 Å).

1.2. Nuclear Magnetic Spectroscopy (NMR)

The nuclear magnetic resonance spectra of ^{1}H NMR measurements were recorded in $CDCl_3$ (deuterium chloroform) operating under a static field strength of 400MHz was recorded on a Bruker spectrometer (Bruker, AVANCE III HD) with $CDCl_3$ (99.8%, Aldrich) as a solvent.

2. Results and Discussions

2.1. Thermal Analysis

Figure S1 shows thermograms (TGA) of (a) PCL-g-MA produced for various reaction pe-riods, (b) with various amounts of MA added and (c) with various amounts of DBPO added.

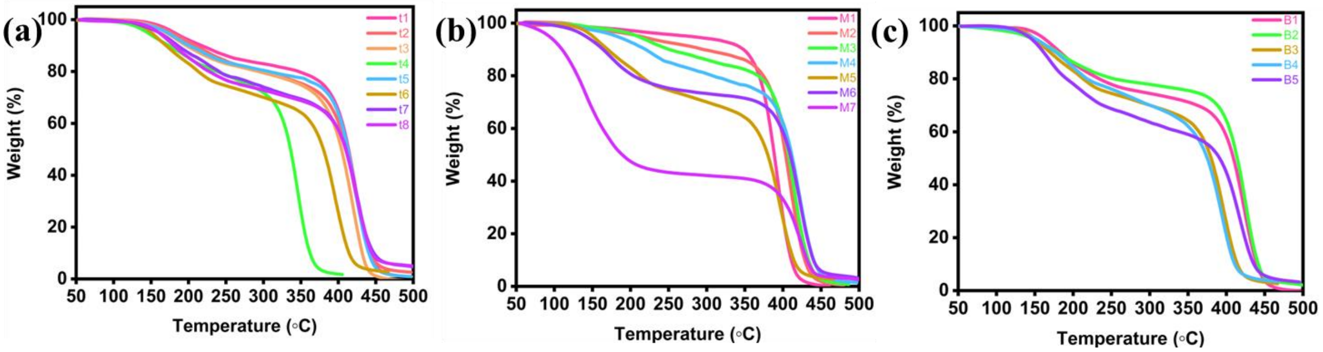

Figure S1. Thermograms of PCL-g-MA produced for (a) various reaction periods, with (b) various amounts of MA added, and with (c) various amounts of DBPO added.

2.2. Derivative thermogravimetric analysis (DTG)

Figure S2 compares DTG curves of PCL-g-MA produced in various reaction conditions. PCL-g-MA exhibits two weight loss steps: MA degradation 106–188 °C and PCL degradation 282–430 °C (Fig.A2a–c). PCL-g-MA by various amounts of MA added decreased in the initial decomposition temperature with the increased of MA added because free MA was bound to PCL radical sites during this reaction [1] (Fig.S2b). PCL-g-MA by various amounts of MA added (Fig.S2b) shows the same trend as the experimental designs by various DBPO added (Fig.S2c). All the grafted samples underwent more weight losses than PCL because of the degradation of the MA portion. In comparison with PCL, PCL-g-MA produced in each condition has lower thermal stability. Because the peroxide leads to scission of the macromolecular chains to generate the PCL radicals, MA reacts with PCL and turns out in lower stability [2].

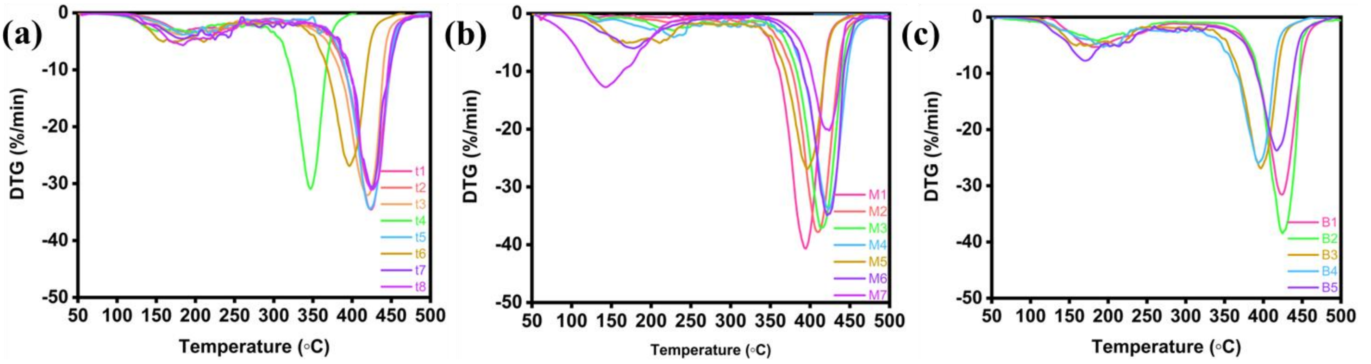

Figure S2. Derivative thermogravimetric analysis (DTG) of PCL-g-MA produced for (a) various reaction periods, with (b) various amounts of MA added, and with (c) various amounts of DBPO added.

2.3. Differential Scanning Calorimetry (DSC)

Figure S3c and Table S2 show DSC curves of PCL-g-MA produced for various reaction periods compared with those of plain PCL (Figure S3a and Table S1). Cold crystallization was observed as a convex peak upward appearing at approx. 20°C, i.e., cold crystallization temperature (T_c) at the top half of the graph and the heat of the cold crystallization (ΔH_c) corresponds to an area of the peak. The enthalpy of melting (ΔH_m) calculated as an area of a convex peak downward appearing at approx. 60 °C at the bottom half of the graph decreased with an increased reaction time and the melting temperature (T_m) decreased as well presumably due to a decrease in the coagulation power among PCL molecules by introduction of slightly hydrophilic groups.

Table S1 Summary of DSC measurement results of plain PCL.

Sample	T_m (°C)	ΔH_m (mJ/mg)	T_c (°C)	ΔH_c (mJ/mg)	X_c (%)
PCL	68.4	153.2	21.5	82.44	59.3

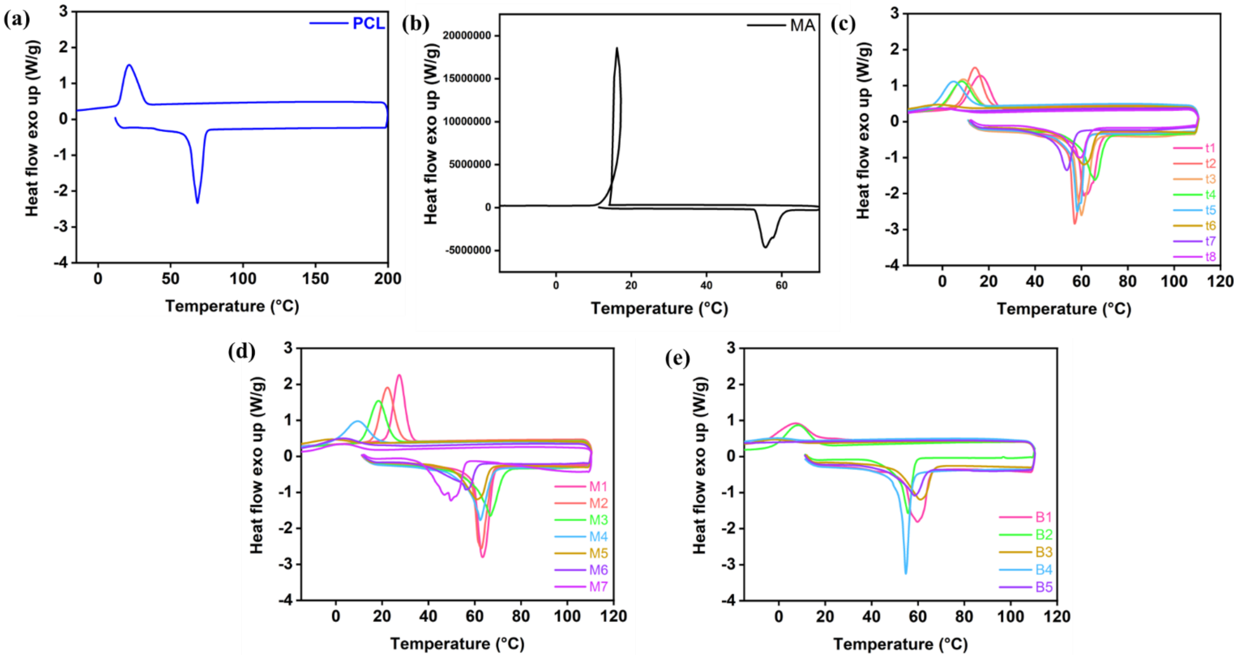

Figure S3. Differential scanning calorimetry (DSC) thermograms (total heat flow exo up) of (a)PCL, (b) MA, PCL-g-MA produced for (c) various reaction periods, with (d) various amounts of MA added, and with (e) various amounts of DBPO added.

Table S2 Summary of DSC measurement results of PCL-g-MA produced for various reaction periods.

Sample	T_m (°C)	ΔH_m (mJ/mg)	T_c (°C)	ΔH_c (mJ/mg)	X_c (%)
t1	61.1	127.98	16.4	62.93	45.2
t2	57.0	137.73	14.1	57.94	41.7
t3	59.9	128.05	9.0	55.88	40.2
t4	65.7	99.07	8.2	55.15	40.0
t5	58.1	113.50	4.8	51.12	36.8
t6	60.9	62.83	1.7	16.13	11.6
t7	53.6	56.10	-1.2	-11.00	N/A
t8	59.4	52.50	-1.2	-17.80	N/A

Table S3 Summary of DSC measurement results of PCL-g-MA produced with various amounts of MA added.

Sample	T_m (°C)	ΔH_m (mJ/mg)	T_c (°C)	ΔH_c (mJ/mg)	X_c (%)
M1	63.5	146.5	27.3	70.8	50.9
M2	62.8	148.0	22.1	69 .0	49.6
M3	66.7	138.7	18.5	60.7	43.7
M4	62.4	125.6	9.3	52.2	37.6
M5	60.9	62.8	1.7	16.1	11.6
M6	56.2	54.0	3.1	-21.8	N/A
M7	49.7	77.9	3.0	-29.6	N/A

Figure S3d shows DSC curves of PCL-g-MA with various amounts of MA added. Table S3 summarizes T_c and ΔH_c of those samples and suggests that more addition of MA more decreased T_c and ΔH_c, corresponding to more grafting of MA on principal PCL chains [3].

Fig.A3e and Table A1.4 show DSC curves of PCL-g-MA with various amounts of DBPO added. Table A1.4 summarizes Tc and ΔHc of those samples and suggests that more addition of DBPO more decreased Tc and ΔHc, corresponding to more grafting of MA on principal PCL chains ΔHc and Tc of various DBPO added. The increase of DBPO contents increasing at the same level of MA concentration and the same time promote a decrease in ΔHc and Tc of synthesized PCL-g-MA at various amounts of DBPO added in comparison with the ΔHc and Tc of PCL [2].

Table S4 Summary of DSC measurement results of PCL-g-MA produced with various amounts of DBPO added.

Sample	T_m (°C)	ΔH_m (mJ/mg)	T_c (°C)	ΔH_c (mJ/mg)	X_c (%)
B1	59.7	134.4	7.1	56.2	40.4
B2	55.8	60.3	8.4	47.4	34.1
B3	60.9	62.8	1.7	16.1	11.6
B4	54.7	123.4	-0.7	7.1	5.1
B5	58.4	87.9	N/A	N/A	N/A

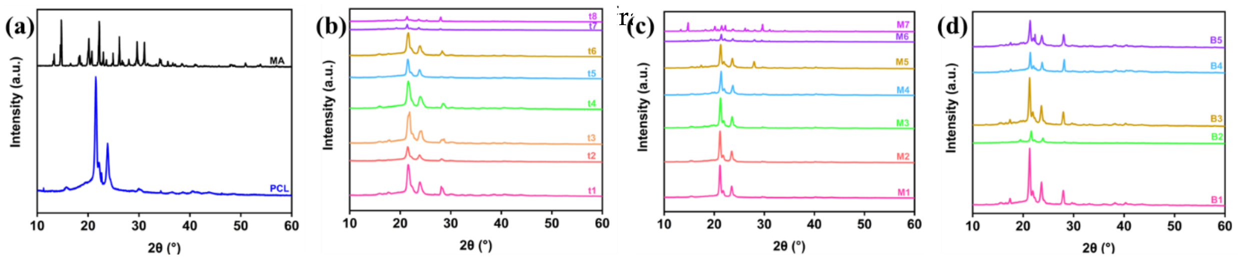

Figure S4. X-ray Diffraction (XRD) of (a) PCL and MA, PCL-g-MA produced for (b) various reaction periods, (c) with various amounts of MA added and (d) with various amounts of DBPO added.

Fig. A4 shows XRD patterns of all the PCL-g-MA samples. Both plain PCL, and MA-grafted PCL samples have three distinctive diffraction peaks observed at 2θ degrees of approx. 21.55°, 22.25° and 23.88°, corresponding to the crystal lattice planes: (110), (111) and (200), respectively. For the MA-grafted PCL samples in every condition, the amorphous halo is observed with the peak at 21.55°, therefore a small displacement of the peak could have occurred, indicating small changes in the interplanar basal spacing with the different condition of grafted PCL [4].

2.5. Nuclear Magnetic Resonance Spectroscopy (NMR)

Figure S5 shows ^{1}H NMR spectra of PCL-g-MA, MA and PCL in $CDCl_3$. A residual solvent peak originating from $CDCl_3$ is found at 7.26 ppm [5-7]. The ^{1}H NMR spectrum of PCL ($CDCl_3$, δ, ppm): (a) 4.03 [2H, t, $–C(O)OCH_2CH_2–$], (e) 2.28 [2H, t, $–CH_2CH_2CH_2CH_2CH_2COO–$], (c) 1.37 [2H, m, $–CH_2CH_2CH_2CH_2CH_2COO–$], (b, d) 1.62 [4H, m, $–CH_2CH_2CH_2CH_2CH_2COO–$]. The ^{1}H NMR spectrum at 7.04 ppm indicates the H proton from the Maleic anhydride structure. The result of the ^{1}H NMR spectrum of PCL-g-MA indicates the characteristic signal at 7.04 ppm (H proton) from Maleic anhydride and the ^{1}H NMR spectrum (a-e) of PCL.

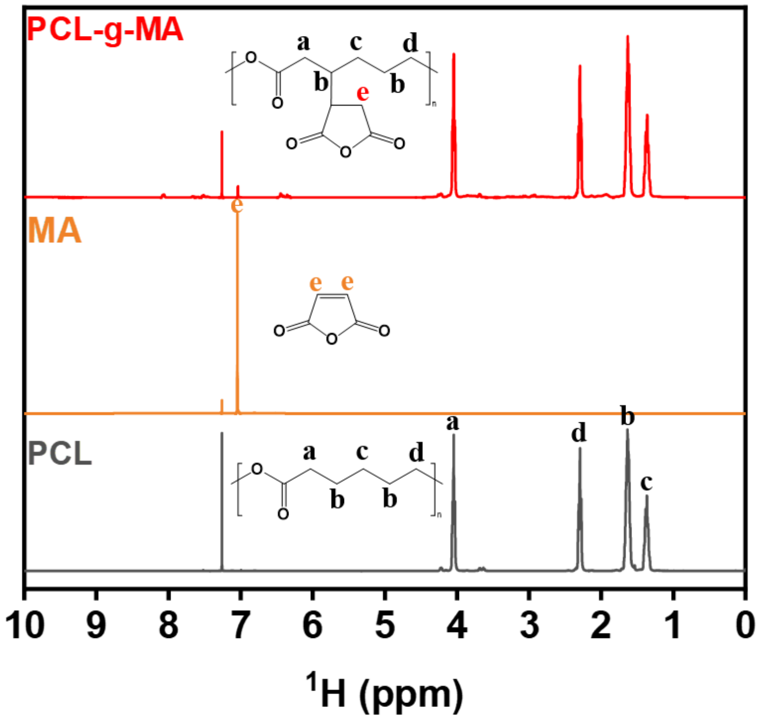

Figure S5. Nuclear Magnetic Resonance (NMR) of (a) PCL, (b) MA, and (c) PCL-g-MA.

2.6. X-ray photoelectron spectroscopy (XPS)

Table S5 Summary of the high-resolution results of PCL and MA.

Samples	C1s			O1s		
	C–C	C–O	C=O	C–O	C=O	C=O (amide unit)
PCL	284.5	286.3	288.5	535.0	533.7	-
MA	286.7	288.2	290.6	535.2	533.7	534.5

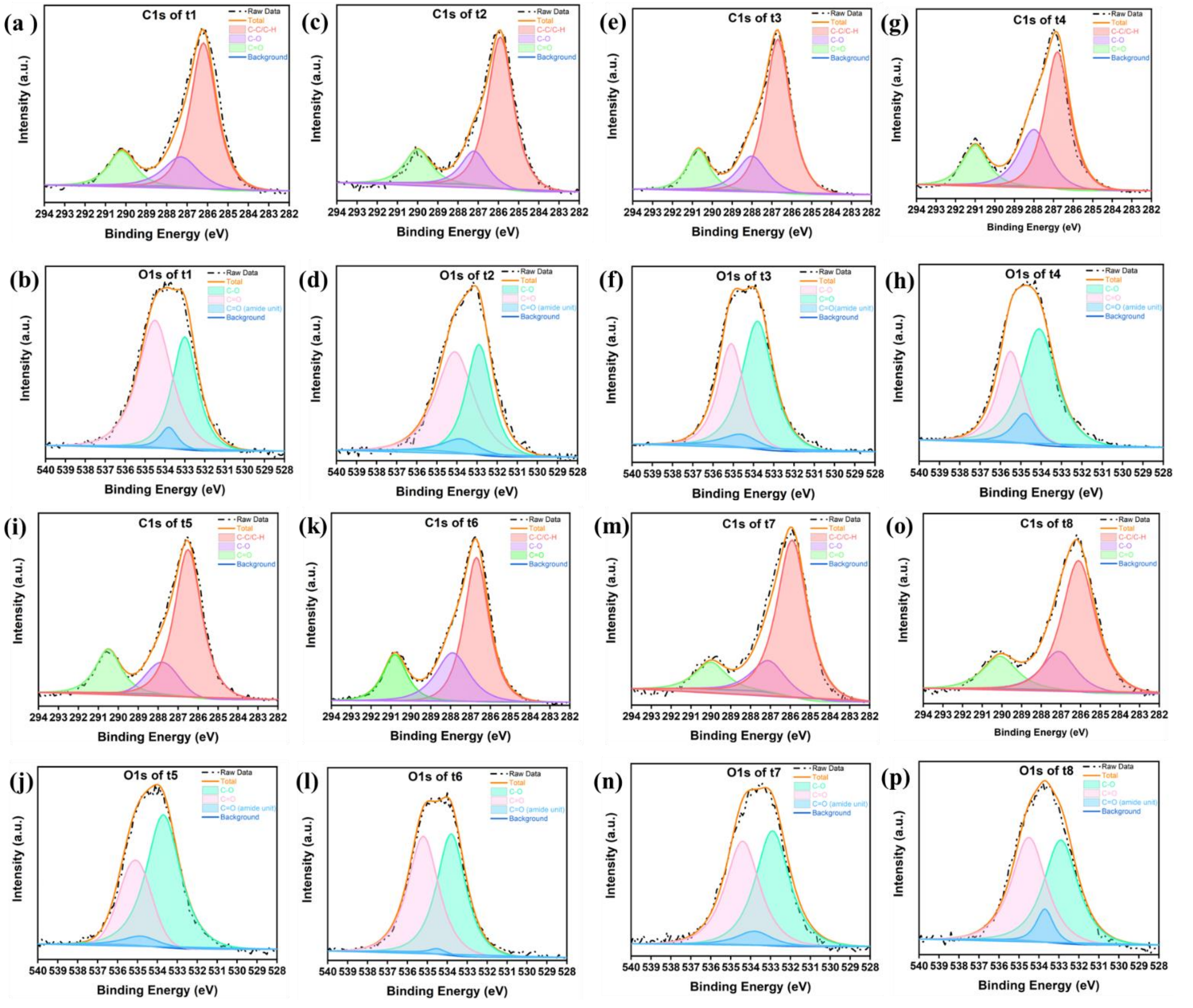

Figure S6. High resolution XPS spectra for C1s and O1s of various reaction time (a-b) t1, (c-d) t2, (e-f) t3, (g-h) t4, (i-j) t5, (k-l) t6, (m-n) t7 and (o-p) t8.

Table S6. Summary of the high-resolution results of PCL-g-MA by various times.

Sample	C1s			O1s		
	C-C	C-O	C=O	C-O	C=O	C=O (amide unit)
t1	290.2	287.3	286.2	534.5	533.0	533.8
t2	290.0	287.2	285.9	534.1	532.9	533.8
t3	290.7	288.0	286.7	535.1	533.8	534.6
t4	291.0	288.0	286.8	535.5	534.1	534.8
t5	290.5	287.8	286.5	535.1	533.7	534.8
t6	290.8	288.0	286.8	535.2	533.8	534.5
t7	290.0	287.1	285.9	534.4	532.9	533.8
t8	290.1	287.1	286.1	534.5	532.9	533.7

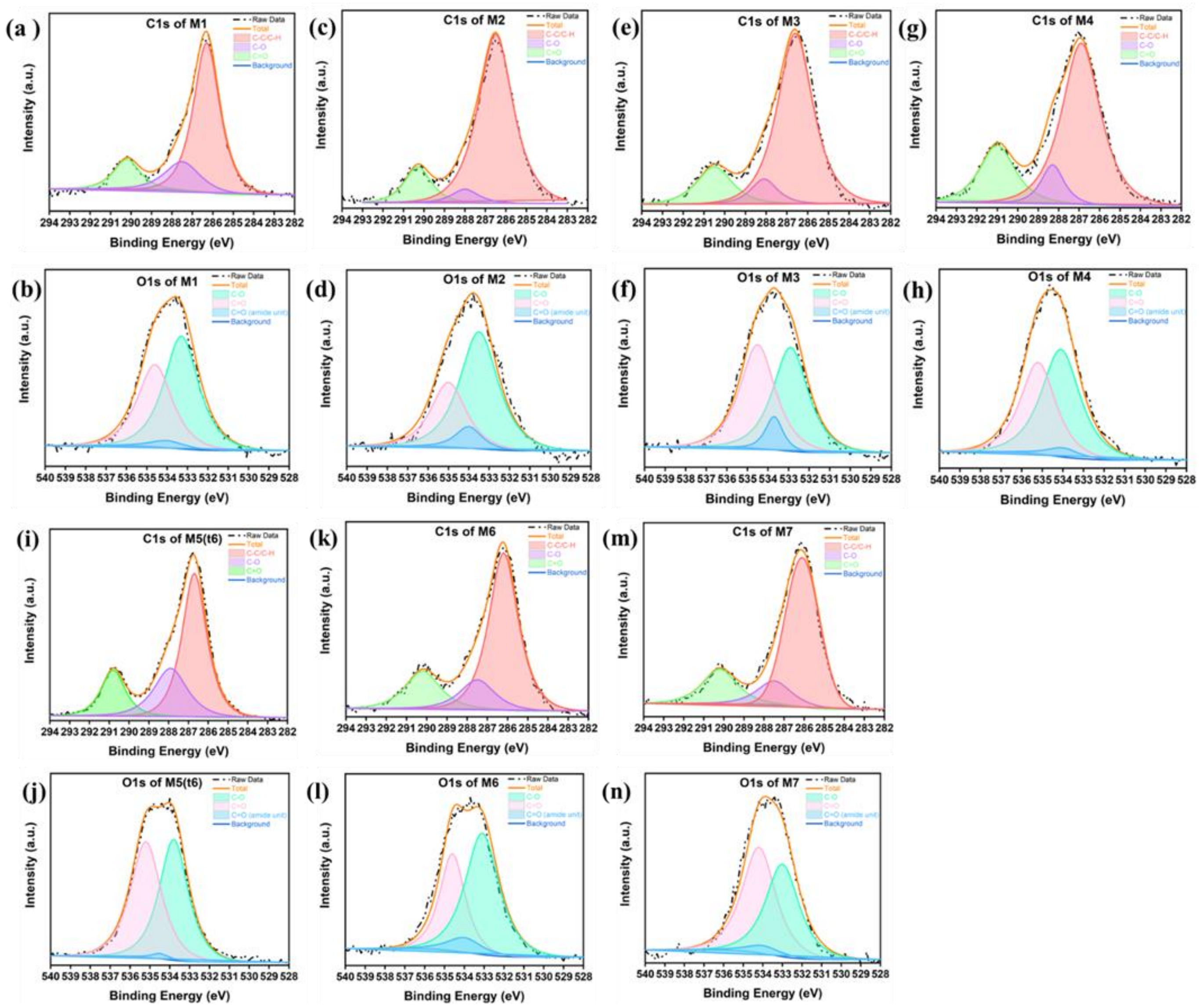

Figure S7. High resolution XPS spectra for C1s and O1s of various MA added (a-b) M1, (c-d) M2, (e-f) M3, (g-h) M4, (i-j) M5, (k-l) M6 and (m-n) M7.

Table S7 Summary of the high-resolution results of PCL-g-MA by various MA added.

Samples	C1s			O1s		
	C-C	C-O	C=O	C-O	C=O	C=O (amide unit)
M1	290.2	287.5	286.3	534.6	533.3	534.0
M2	290.3	288.0	286.5	535.0	533.5	534.0
M3	290.5	288.1	286.6	535.0	533.6	534.0
M4	291.0	288.3	286.9	535.2	534.1	534.0
M5	290.8	288.0	286.8	535.2	533.8	534.5
M6	290.2	287.5	286.2	533.1	534.6	534.0
M7	290.2	287.5	286.1	533.0	534.2	534.0

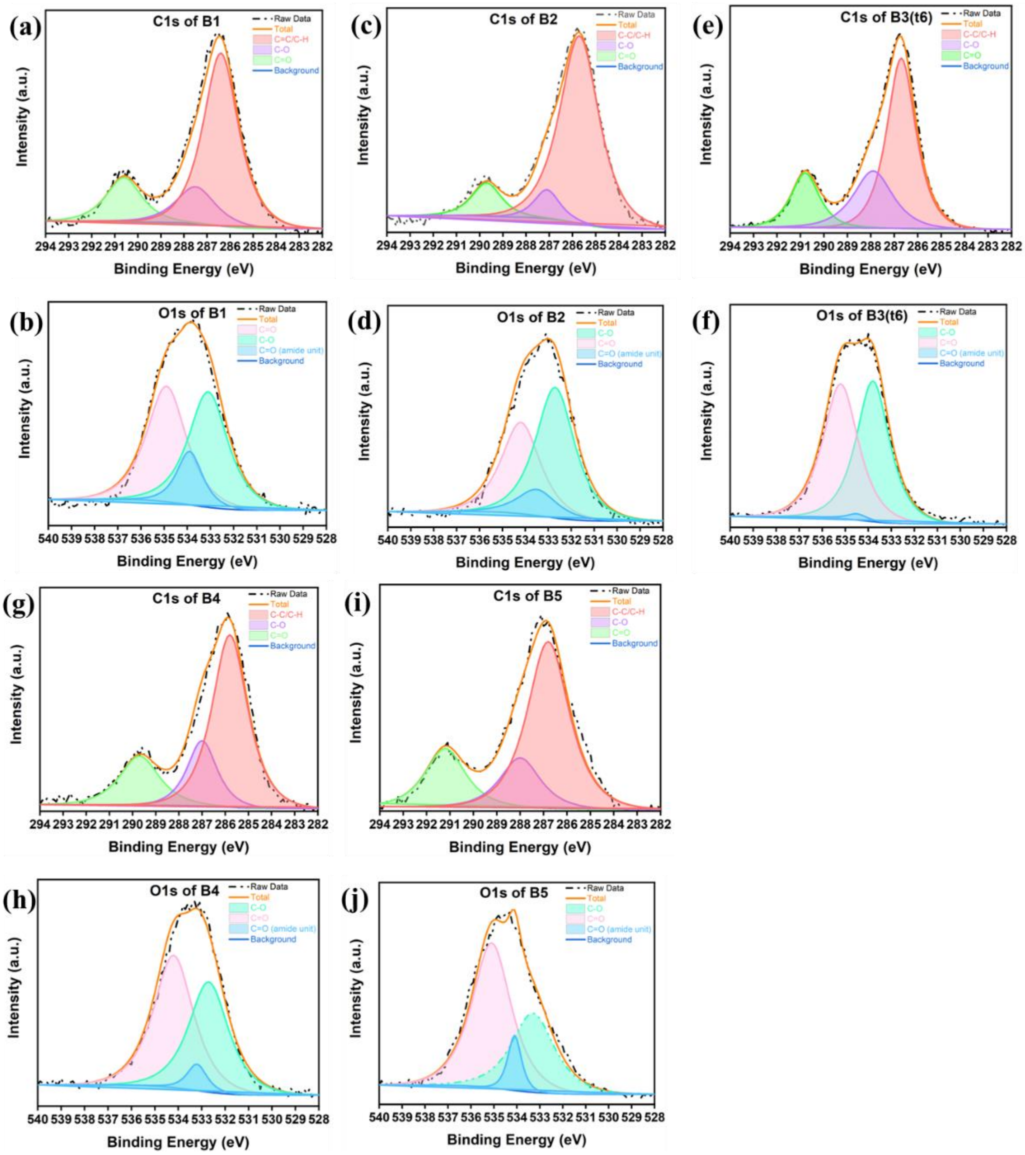

Figure S8. High resolution XPS spectra for C1s and O1s of various DBPO added (a-b) B1, (c-d) B2, (e-f) B3, (g-h) B4 and (i-j) B5

Table S8 Summary of the high-resolution results of PCL-g-MA by various DBPO added.

Samples	C1s			O1s		
	C-C	C-O	C=O	C-O	C=O	C=O (amide unit)
B1	290.6	287.5	286.4	533.1	534.9	533.9
B2	289.7	287.1	285.7	532.7	534.2	533.5

B3	290.8	288.0	286.8	535.2	533.8	534.5
B4	289.7	287.0	285.8	532.7	534.2	533.2
B5	291.2	288.0	286.8	533.3	535.1	534.1

Table S9. Summary of the Ti, Tmax, Tf, DTG at main Tmax peak and residue at 550 °C at the different heating rates.

**Heating rate (°C/min)**	**1st step**				**2nd step**				**Residue at 550 °C**
	T_i	T_{max}	T_f	*DTG at main T_{max} peak*	T_i	T_{max}	T_f	*DTG at main T_{max} peak*	
**5**	78	162	242	24.3	300	396	463	72.1	3.62
**10**	88	167	249	28.9	315	404	468	65.6	5.49
**15**	108	180	262	23.2	332	420	470	70.9	5.86
**20**	119	180	285	27.6	332	425	482	66.6	5.86
**25**	120	194	293	23.5	350	431	483	70.0	6.55
**30**	121	196	297	23.5	352	435	493	70.0	6.55

Table S10. Activation energy calculated based on the contracting volume model (R3) at various heating rates (8.314 applied as the gas constant R).

**Heating rate (°C/min)**	**c**	**Slope (m)**	**E_a (J/mol)**	**R^2**
**5**	**-14.2**	**-737.07**	**-88.65**	0.9949
**10**	-14.2	-730.02	-87.81	0.9882
**15**	-14.2	-718.56	-86.43	0.9839
**20**	-14.2	-718.09	-86.37	0.9822
**25**	-14.2	-711.68	-85.60	0.9747
**30**	-14.2	-709.98	-85.40	0.9706

References

1. Ortega-Toro, R.; Santagata, G.; Gomez d'Ayala, G.; Cerruti, P.; Talens Oliag, P.; Chiralt Boix, M.A.; Malinconico, M. Enhancement of interfacial adhesion between starch and grafted poly(ε-caprolactone). *Carbohydr. Polym.* **2016**, *147*, 16-27.
2. Lopera-Valle, A.; Elias, A. Amine Responsive Poly (lactic acid)(PLA) and Succinic Anhydride (SAh) Graft-Polymer: Synthesis and Characterization. *Polymers* **2019**, *11,* 1466.
3. Mani, R.; Bhattacharya, M.; Tang, J. Functionalization of polyesters with maleic anhydride by reactive extrusion. *J Polym. Sci. A. Polym. Chem.* **1999**, *37*, 1693-1702.
4. Morais, D.; Siqueira, D.; Bruno Barreto Luna, C.; Araujo, E.; Bezerra, E.; Wellen, R. Grafting Maleic Anhydride onto Polycaprolactone: Influence of processing. *Mater. Res. Express* **2019**, *6*, 055315.
5. Haque, M.; Errico, M.; Gentile, G.; Avella, M.; Pracella, M. Functionalization and compatibilization of poly(ε-caprolactone) composites with cellulose microfibres. *Macromol. Mater. Eng.* **2012**, *297,* 985-993.
6. Fulmer, G.R.; Miller, A.J.M.; Sherden, N.H.; Gottlieb, H.E.; Nudelman, A.; Stoltz, B.M.; Bercaw, J.E.; Goldberg, K.I. NMR Chemical Shifts of Trace Impurities: Common Laboratory Solvents, Organics, and Gases in Deuterated Solvents Relevant to the Organometallic Chemist. *Organometallics* **2010**, *29*, 2176-2179.
7. Khankrua, R.; Pivsa-Art, S.; Hiroyuki, H.; Suttiruengwong, S. Grafting of poly (lactic acid) with maleic anhydride using supercritical carbon dioxide. *IOP Conf. Ser.: Mater. Sci. Eng.* **2015**, *87*, 012066.
